# Adjunctive Synbiotic Therapy for *Helicobacter pylori* Eradication: An Updated Systematic Review and Meta-Analysis of Randomized Controlled Trials

**DOI:** 10.3390/life16091409

**Published:** 2026-08-25

**Authors:** Chonticha Romyasamit, Thitinat Duangchan, Yosita Leepromma, Morteza Saki, Phoomjai Sornsenee

**Affiliations:** 1 Department of Medical Technology, School of Allied Health Sciences, Walailak University, Thasala 80160, Nakhon Si Thammarat, Thailand; chonticha.ro@wu.ac.th (C.R.); thitinat.du@wu.ac.th (T.D.); yosita.le@mail.wu.ac.th (Y.L.); 2 Research Center for Microbiome, Systems Biology, and Medical Innovation, Walailak University, Thasala 80160, Nakhon Si Thammarat, Thailand; 3 Research Center in Tropical Pathobiology, Walailak University, Thasala 80160, Nakhon Si Thammarat, Thailand; 4 Department of Microbiology, Faculty of Medicine, Ahvaz Jundishapur University of Medical Sciences, Ahvaz, Iran; mortezasaki1981@gmail.com; 5 Department of Family and Preventive Medicine, Faculty of Medicine, Prince of Songkla University, Hat Yai 90110, Songkhla, Thailand

**Keywords:** *Helicobacter pylori*, synbiotics, eradication therapy, adverse events, meta-analysis

## Abstract

Background: Synbiotics may improve the effectiveness of antibiotic-based *Helicobacter pylori* eradication therapy; however, the available evidence remains limited. This systematic review and meta-analysis updated the evidence on the efficacy and safety of adjunctive synbiotics for *H. pylori* eradication and treatment-related adverse events. Methods: This systematic review and meta-analysis followed the Preferred Reporting Items for Systematic Reviews and Meta-Analyses (PRISMA 2020) guidelines and was registered in PROSPERO (CRD420251236314). PubMed, Scopus, Embase, MEDLINE, Web of Science, and Google Scholar were searched from inception to their respective final search dates in November and December 2025 for randomized controlled trials comparing antibiotic-based eradication therapy plus a synbiotic with the corresponding regimen without synbiotics. Risk ratios (RRs) with 95% confidence intervals (CIs) were pooled for intention-to-treat (ITT), per-protocol (PP), and safety outcomes. Risk of bias was assessed using the Cochrane Risk of Bias 2 tool, and certainty of evidence was evaluated using the GRADE approach. Results: Six randomized controlled trials enrolling 486 participants were included; 446 participants contributed to the ITT analysis and 416 to the PP analysis. One trial was judged to be at high risk of bias, whereas five raised some concerns. The ITT analysis demonstrated improved eradication with adjunctive synbiotics (RR 1.18, 95% CI 1.04–1.34; *I*^2^ = 44.53%). No significant benefit was observed in the PP analysis (RR 1.06, 95% CI 0.95–1.18; *I*^2^ = 48.40%). Safety data were available from three pediatric trials (*n* = 230), with low-certainty evidence suggesting fewer overall adverse events (RR 0.27, 95% CI 0.16–0.45). Conclusions: Adjunctive synbiotics may modestly improve *H. pylori* eradication and may reduce overall adverse events. However, the eradication benefit was not confirmed in PP analyses, and the safety findings should be interpreted cautiously given the limited pediatric evidence.

## 1. Introduction

*Helicobacter pylori* is a Gram-negative, spiral-shaped, microaerophilic bacterium capable of persistently colonizing the gastric mucosa [1]. It remains one of the most prevalent chronic bacterial infections worldwide, with a recent global analysis estimating a prevalence of 43.9% among adults during 2015–2022 [2]. Long-term colonization induces chronic inflammation of the gastric epithelium, leading to epithelial damage and disruption of normal gastric function. *H. pylori* infection is associated with a spectrum of upper gastrointestinal disorders, including chronic gastritis, peptic ulcer disease, and mucosa-associated lymphoid tissue lymphoma, and is a major contributor to gastric carcinogenesis [3]. In recognition of its oncogenic potential, *H. pylori* has been classified as a Group I carcinogen by the International Agency for Research on Cancer [4], whereas the World Health Organization has identified clarithromycin-resistant *H. pylori* as a high-priority pathogen because of the growing threat of antimicrobial resistance [5]. Therefore, successful eradication of *H. pylori* has become a major strategy for reducing the incidence of gastric cancer and recurrent peptic ulcer worldwide.

Eradication of *H. pylori* is essential for preventing gastric adenocarcinoma and reducing peptic ulcer recurrence. Clarithromycin-based triple therapy has been widely used as a first-line treatment; however, its effectiveness has declined globally, with eradication rates frequently falling below the accepted threshold of 80% [6]. This decline is primarily attributed to the increasing prevalence of antimicrobial resistance, particularly to clarithromycin, metronidazole, and levofloxacin [7].

Current clinical guidelines recommend alternative regimens, including bismuth-based quadruple, sequential, and concomitant therapies [8]. Nevertheless, eradication rates remain variable, ranging from 60% to 90% across different populations and treatment settings [9]. Moreover, treatment-related adverse events occur in approximately 20–30% of patients receiving *H. pylori* eradication therapy and may negatively affect treatment adherence [10]. Poor treatment adherence resulting from antibiotic-associated adverse events is a major contributor to eradication failure. Therefore, interventions that improve treatment tolerability may indirectly enhance eradication success in routine clinical practice. Common adverse effects, such as diarrhea and gastrointestinal discomfort, may reduce adherence and lead to premature discontinuation of therapy [11]. However, despite these advances, no universally effective regimen has been established, highlighting the need for adjunctive strategies, such as synbiotics, to improve eradication rates and reduce adverse effects [12].

According to the International Scientific Association for Probiotics and Prebiotics (ISAPP), synbiotics are mixtures of live microorganisms and substrates selectively utilized by host microorganisms that confer health benefits. Unlike probiotics, synbiotics have synergistic effects by enhancing probiotic survival, colonization, and metabolic activity through complementary prebiotic substrates [13]. They have emerged as a promising adjunctive strategy for improving *H. pylori* eradication [12]. Probiotics, particularly *Lactobacillus* [14] and *Bifidobacterium* spp. [15] exhibit anti-*H. pylori* activity through multiple mechanisms, including production of bacteriocins and organic acids, inhibition of bacterial adhesion to gastric epithelial cells, and modulation of host immune responses [16]. Prebiotics, such as fructooligosaccharides and galactooligosaccharides, selectively stimulate the growth and metabolic activity of beneficial microbiota, thereby enhancing probiotic survival and function [17]. The synergistic interaction between probiotics and prebiotics may help maintain the gastrointestinal microbial balance during antibiotic therapy, reduce treatment-related adverse effects, and ultimately improve eradication outcomes [18].

Although the adjunctive use of probiotics has been extensively investigated and supported by several systematic reviews and meta-analyses, evidence specifically evaluating synbiotics remains limited. The only previous systematic review focusing exclusively on synbiotics included six randomized controlled trials published up to 2018 [19].

Since then, another randomized controlled trial of adjunctive synbiotic therapy with a current bismuth-based quadruple eradication regimen has been published [20]. Moreover, the previous review did not incorporate contemporary evidence synthesis standards, including the Preferred Reporting Items for Systematic Reviews and Meta-Analyses (PRISMA 2020) guidelines, the Cochrane Risk of Bias 2 (RoB 2) tool, and the Grading of Recommendations Assessment, Development and Evaluation (GRADE) approach. Therefore, an updated and methodologically rigorous systematic review and meta-analysis are needed to provide a comprehensive and contemporary assessment of the efficacy and safety of adjunctive synbiotic therapy for *H. pylori* eradication.

Therefore, we conducted an updated systematic review and meta-analysis of randomized controlled trials to comprehensively evaluate the efficacy and safety of adjunctive synbiotic therapies for *H. pylori* eradication. In addition to pooled analyses of eradication outcomes and adverse events, we assessed study quality using the RoB 2 tool and evaluated the certainty of evidence using the GRADE approach to provide an updated evidence base for clinical practice and future guideline development.

## 2. Materials and Methods

### 2.1. Study Registration

This systematic review and meta-analysis was conducted in accordance with the PRISMA 2020 guidelines [21]. The study protocol was registered in the International Prospective Register of Systematic Reviews (PROSPERO; CRD420251236314) on 21 November 2025. Registration occurred after the initial searches of PubMed, Scopus, Embase, and MEDLINE, but before the Web of Science and Google Scholar searches and before data extraction, risk-of-bias assessment, and statistical analysis were completed.

### 2.2. Search Strategy

A comprehensive literature search was conducted using PubMed, Scopus, Embase, MEDLINE, Web of Science, and Google Scholar from database inception to the respective search dates. PubMed, Scopus, Embase, and MEDLINE were searched on 14 November 2025. Web of Science and Google Scholar were searched on 19 December 2025. No publication-date restriction was applied, and no language restriction was applied at the search stage. The search strategy comprised two main concepts: *H. pylori* infection and synbiotic intervention. Controlled vocabulary terms, including Medical Subject Headings and Emtree terms, were combined with the free-text keywords. Boolean operators, database-specific truncations, and field tags were applied as appropriate. Google Scholar results were sorted by relevance, and the first 210 records were screened. This cut-off was used because relevance declined substantially after approximately 200 records, with no potentially eligible randomized trials identified among the final screened records. Trial registries and dedicated grey literature databases were not searched. Full database-specific search strategies and the number of records retrieved are presented in Supplementary Table S1.

### 2.3. Study Selection

All retrieved records were imported into Rayyan (Rayyan Systems Inc., Cambridge, MA, USA), and duplicate records were identified and removed. Two reviewers (C.R. and Y.L.) independently screened the titles and abstracts of the remaining records. Reports considered potentially eligible were retrieved in full text and independently assessed against the predefined eligibility criteria. Disagreements at either stage were resolved by a third reviewer (T.D.). Studies were eligible if they were randomized controlled trials that enrolled participants with confirmed *H. pylori* infection and compared standard antibiotic-based eradication therapy plus a synbiotic intervention with the same or an equivalent eradication regimen without synbiotic supplementation. Studies were excluded if they used a nonrandomized or otherwise ineligible study design; evaluated synbiotics without concomitant antibiotic-based eradication therapy; investigated probiotics or prebiotics alone; used an ineligible comparator; or did not report eligible or extractable eradication outcomes. Reviews, case reports, conference abstracts without sufficient data, trial registration records, letters to the editor, animal or laboratory studies, and other non-original publications were also excluded.

### 2.4. Data Extraction

Two reviewers (C.R. and Y.L.) independently extracted data using a standardized Microsoft Excel (Microsoft Corporation, Washington, DC, USA) form. The extracted information included the first author, publication year, country, study design, sample size, participant age and sex, diagnostic method used to confirm *H. pylori* infection, and timing of outcome assessment. Intervention characteristics included the probiotic species and strains, prebiotic components, synbiotic dose and duration, antibiotic-based eradication regimen, and comparator. For each treatment group, the number of participants with successful eradication and the total number analyzed were extracted separately for the intention-to-treat (ITT) and per-protocol (PP) populations. Safety data included the number of participants who experienced at least one adverse event, diarrhea, and nausea and/or vomiting. Disagreements were resolved through discussion and adjudication by a third reviewer (T.D.). For multi-arm trials, only intervention and comparator arms that enabled isolation of the effect adjunctive synbiotic therapy were included. In Wang et al. [20] only the quadruple therapy alone (QT) and quadruple therapy plus synbiotics (QS) arms were extracted; arms involving fucoidan as an additional co-intervention were excluded. Since a control arm was not used for more than one comparison, no unit-of-analysis adjustment was necessary.

All randomized participants in the eligible comparison arms were retained in the denominator for the ITT eradication analysis. Participants without a documented post-treatment eradication result because of loss to follow-up, treatment discontinuation, non-adherence, or other post-randomization exclusions were conservatively classified as treatment failures. ITT and PP event counts were either extracted directly when reported or reconstructed from participant disposition and eradication data provided in the original trial reports. Consequently, where post-randomization exclusions occurred, the number of documented eradication successes remained unchanged between the ITT and PP analyses, whereas the corresponding denominators differed. The extracted study-level dataset used for the ITT, PP, and adverse-event analyses is provided in Supplementary Dataset S1.

### 2.5. Risk of Bias Assessment

Two reviewers (C.R. and Y.L.) independently assessed the risk of bias in each included randomized controlled trial using the RoB 2 tool. The assessment focused on the ITT *H. pylori* eradication outcome and evaluated the effect of assignment to the intervention. Five domains were evaluated: bias arising from the randomization process, bias owing to deviations from intended interventions, bias owing to missing outcome data, bias in the measurement of the outcome, and bias in the selection of the reported result. Each domain and the overall risk of bias were classified as low risk, some concern, or high risk. Disagreements were resolved through discussion and adjudication by a third reviewer (T.D.). Completed RoB 2 signalling questions and supporting rationales for each domain-level judgment are provided in Supplementary Table S2.

### 2.6. Certainty of Evidence

The certainty of evidence for each outcome was assessed using the Grading of Recommendations Assessment, Development and Evaluation (GRADE) approach. Evidence from randomized controlled trials was initially considered to be of high certainty and was downgraded on the basis of risk of bias, inconsistency, indirectness, imprecision, and publication bias. The certainty of evidence was categorized as high, moderate, low, or very low. Summary of findings tables were generated to present the pooled estimates and corresponding certainty ratings.

### 2.7. Statistical Analysis

All statistical analyses were performed using Stata version 19.0 (StataCorp LLC., College Station, TX, USA). Effect estimates were expressed as risk ratios (RRs) with 95% confidence intervals (CIs). *H. pylori* eradication was synthesized separately for intention-to-treat (ITT) and per-protocol (PP) populations using random-effects models with restricted maximum likelihood estimation. When a study contained a zero count in the 2 × 2 table for eradication outcomes, a 0.5 continuity correction was applied for estimation of the study-specific RR and variance. Overall adverse events were pooled using a Mantel–Haenszel fixed-effect model because this approach is commonly used for sparse dichotomous data [22]. Symptom-specific adverse events, including diarrhea and nausea and/or vomiting, were not pooled because events were sparse and several trials contained zero events. Between-study heterogeneity was assessed using the *I*^2^ statistic. *I*^2^ values of 0–25%, >25–50%, >50–75%, and >75% were interpreted as indicating low, moderate, substantial, and considerable heterogeneity, respectively. Leave-one-out sensitivity analyses were conducted for the ITT and PP eradication outcomes by sequentially excluding each study and recalculating the pooled estimates. Subgroup analyses were performed according to the publication period, age group, probiotic composition, prebiotic composition, duration of synbiotic administration, and background eradication regimen. Publication bias could not be reliably assessed because fewer than 10 studies were available for each outcome. Statistical significance was defined as a two-sided *p*-value < 0.05.

## 3. Results

### 3.1. Study Selection

**Figure 1:**
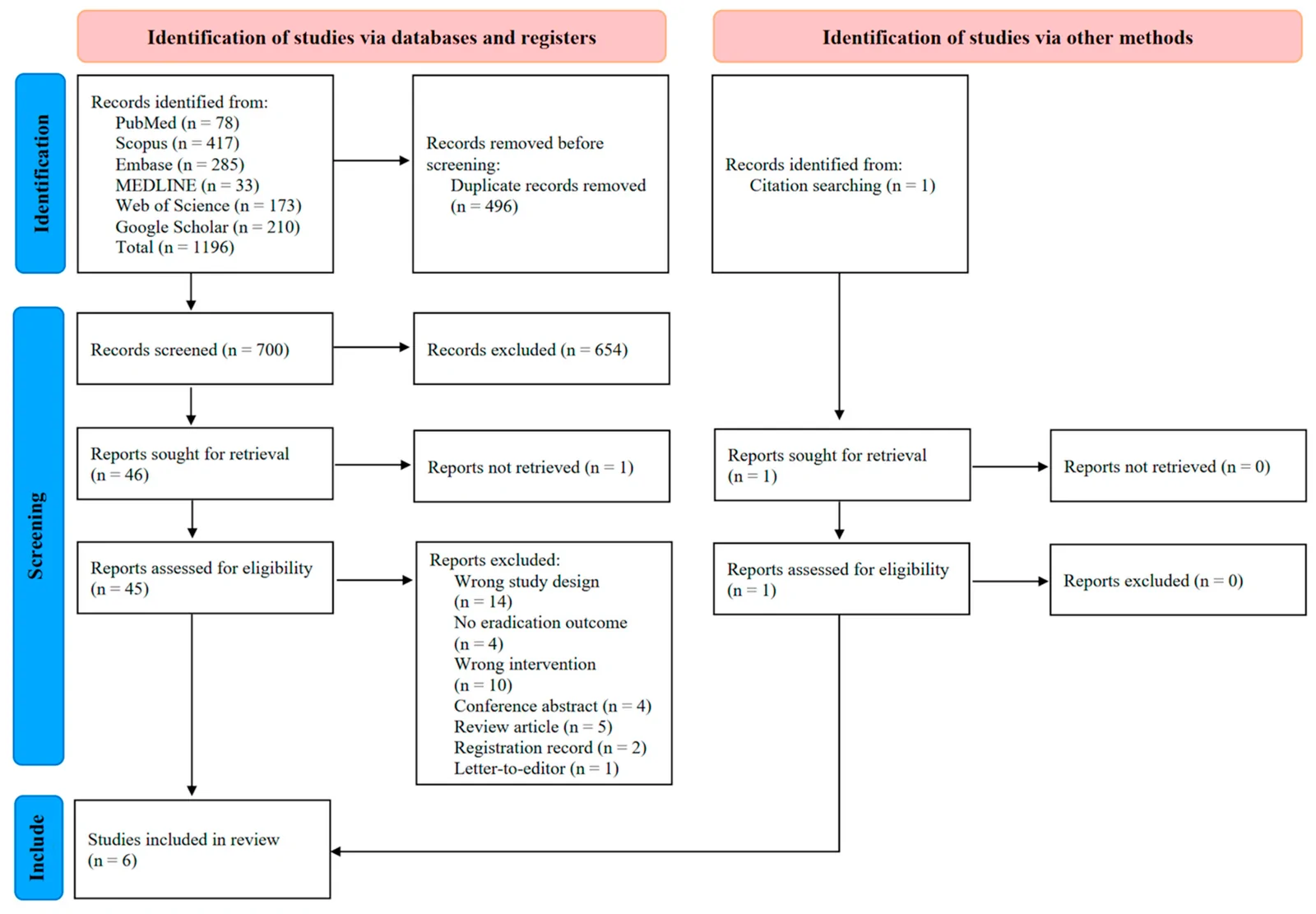
PRISMA flow diagram of study selection.

The database searches identified 1196 records (PubMed, *n* = 78; Scopus, *n* = 417; Embase, *n* = 285; MEDLINE, *n* = 33; Web of Science, *n* = 173; and Google Scholar, *n* = 210). After removing 496 duplicate records, 700 were screened by title and abstract, of which 654 were excluded. Forty-six reports were sought for retrieval, all of which were successfully retrieved and assessed for full-text eligibility. Of these, one report, Şahin et al. [23], could not be retrieved despite additional full-text searching and attempted author contact. Therefore, 45 reports were retrieved and assessed for full-text eligibility. Of these, 40 reports were excluded because of ineligible study design (*n* = 14), absence of an eligible eradication outcome (*n* = 4), ineligible intervention (*n* = 10), publication as a conference abstract (*n* = 4), review article (*n* = 5), registration record (*n* = 2), or letter to the editor (*n* = 1). One additional report, Tolone et al. (2012) [24], was identified through backward citation searching, successfully retrieved, and assessed as eligible. Overall, six studies were included in this systematic review and meta-analysis [20,24,25,26,27,28] (Figure 1).

### 3.2. Characteristics of Included Studies

Six randomized controlled trials published between 2012 and 2024 were included, comprising 486 participants from Turkey, Iran, Italy, and China. Sample sizes ranged from 68 to 100 participants. Four studies enrolled children with mean ages ranging from 8.3 to 12.1 years, whereas two studies enrolled adults with mean ages of approximately 39–44 years. *H. pylori* infection was diagnosed using histology, rapid urease testing, stool antigen testing, and/or urea breath testing, with endoscopic assessment performed in some studies. The synbiotic formulations varied across studies. Three studies evaluated single-strain preparations containing *Bifidobacterium lactis*, whereas three used multi-strain formulations comprising combinations of *Lactobacillus*, *Bifidobacterium*, *Streptococcus*, and other related genera. Inulin was the sole prebiotic component in four studies, whereas one study used fructooligosaccharides and another used a combination of resistant dextrin, fructooligosaccharides, and inulin. Synbiotics were administered for 7 or 14 days in five trials, whereas Wang et al. administered synbiotics for 6 weeks. Four trials evaluated synbiotics as adjuncts to triple eradication therapy, whereas two used bismuth-based quadruple therapy. Detailed study and intervention characteristics are presented in Table 1.

### 3.3. Risk of Bias Assessment of Included Studies

**Figure 2:**
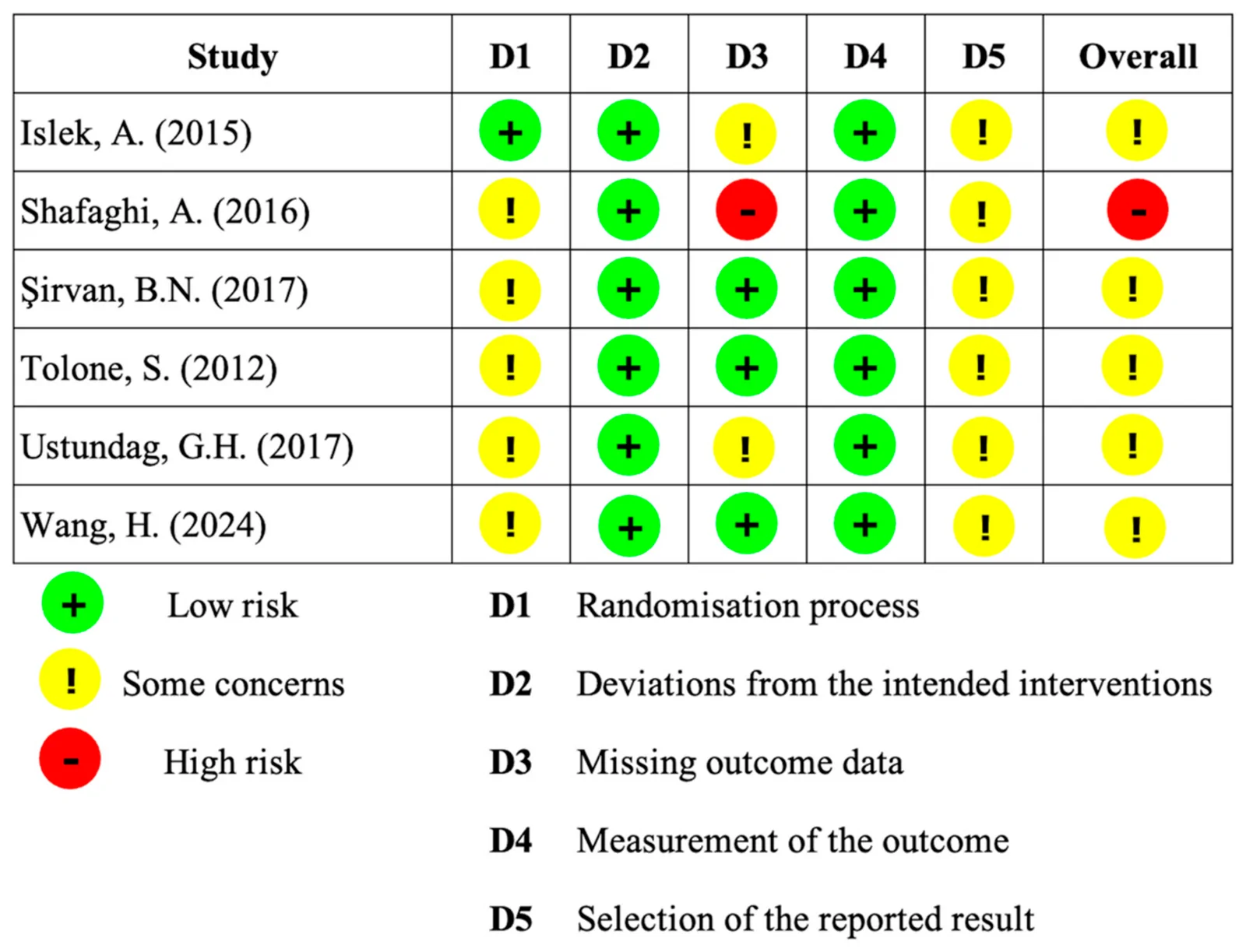
Risk-of-bias assessment of the randomized controlled trials included in the meta-analysis. Risk of bias was evaluated using the Cochrane Risk of Bias 2 (RoB 2) tool for the intention-to-treat (ITT) *Helicobacter pylori* eradication outcome.

Of the six included randomized trials, one was judged to be at high overall risk of bias, and five raised some concerns; none were judged to be at low overall risk. The high-risk judgment for Shafaghi et al. [26] was driven by substantial and differential missing outcome data. The most frequent concerns involved insufficient reporting of the randomization process, allocation concealment procedures, and the absence of an accessible prespecified analysis plan. Some concerns regarding missing outcome data were also identified in the Islek et al. [25] and Ustundag et al. [28] trials. Bias due to deviations from intended interventions and bias in the measurement of the outcome were judged to be at low risk in all studies. For Domain 2, low risk judgments were based on the effect of assignment to intervention; despite the open-label nature of Wang et al. [20], all participants took their assigned therapies and no significant deviations from the intended interventions were noted. Eradication was based on objective diagnostic tests that were applied similarly to both treatment groups in each trial. There was no evidence of differential outcome ascertainment for Domain 4. The domain-level and overall risk-of-bias judgments are presented in Figure 2, and the completed RoB 2 signalling questions and supporting rationales are provided in Supplementary Table S2.

### 3.4. Effect of Synbiotics on H. pylori Eradication

#### 3.4.1. Intention-to-Treat Analysis

**Figure 3:**
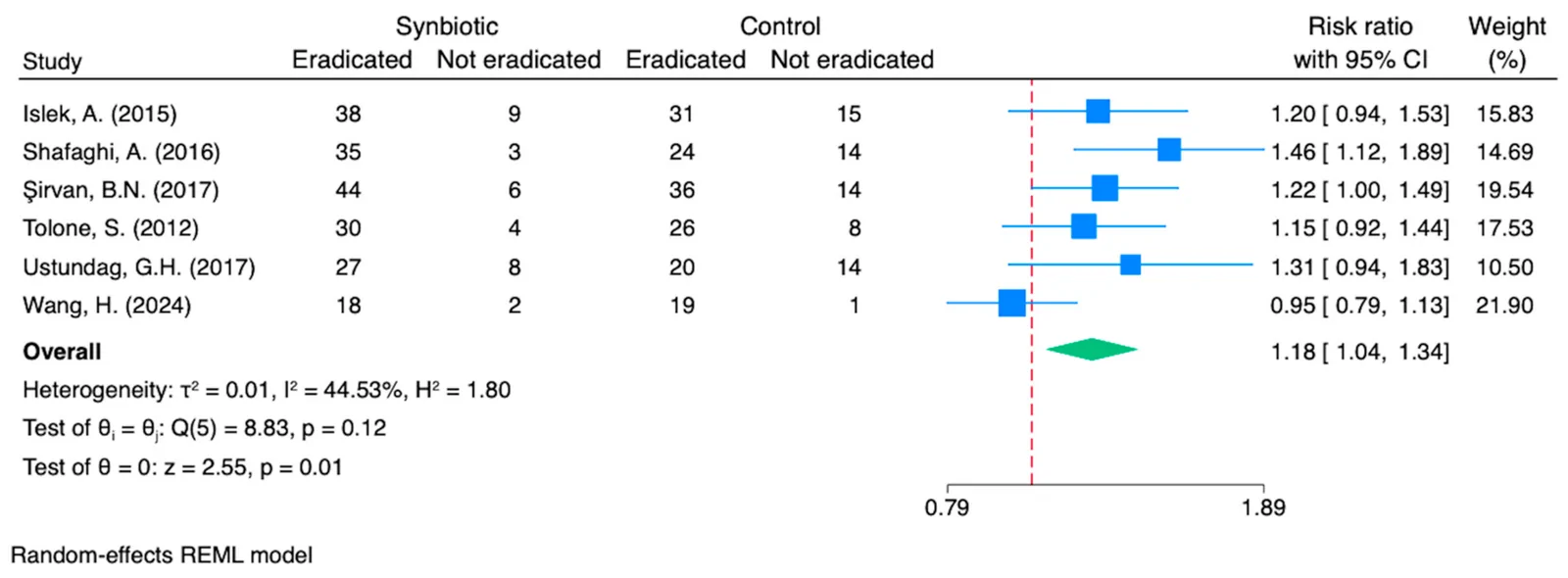
Forest plot of the effect of synbiotic interventions on *Helicobacter pylori* eradication in the intention-to-treat (ITT) analysis.

Six randomized controlled trials involving 446 participants contributed to the ITT analysis. Adjunctive synbiotic therapy was associated with a higher *H. pylori* eradication rate than eradication therapy alone (RR 1.18, 95% CI 1.04–1.34; *p* = 0.011). Moderate between-study heterogeneity was observed (*I*^2^ = 44.53%) (Figure 3). In the leave-one-out sensitivity analysis, the pooled estimates remained statistically significant after sequential omission of each study, ranging from RR 1.13 (95% CI 1.00–1.28) after exclusion of Shafaghi et al. [26] to RR 1.25 (95% CI 1.12–1.39) after exclusion of Wang et al. [20]. Omission of Tolone et al. (25) yielded a similar significant estimate (RR 1.19, 95% CI 1.02–1.39). Although the confidence interval approached the null after removal of the study by Shafaghi et al. [26], no individual study changed the direction or statistical significance of the pooled effect (Supplementary Figure S1). As an additional robustness assessment for the primary ITT outcome, the Hartung–Knapp adjusted confidence interval remained statistically significant but close to the null (RR 1.18, 95% CI 1.01–1.38), whereas the 95% prediction interval (PI) was wide and crossed the null (0.84–1.65).

#### 3.4.2. Per-Protocol Analysis

**Figure 4:**
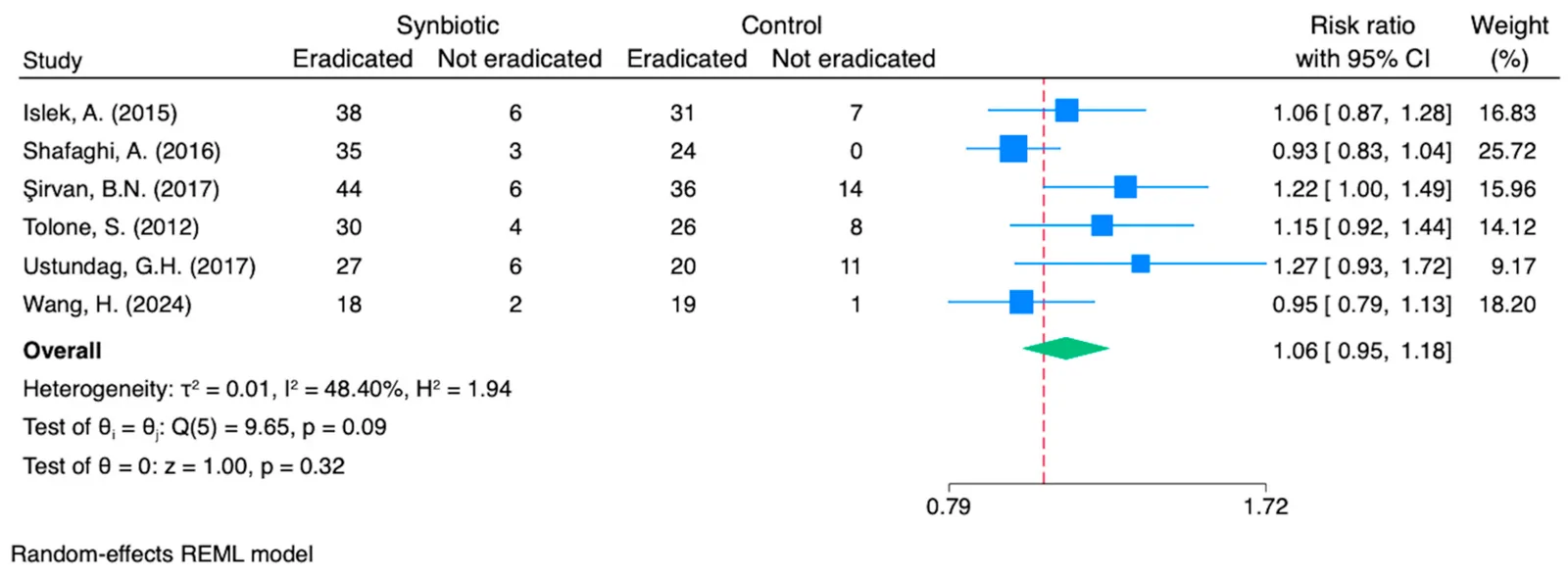
Forest plot of the effect of synbiotic interventions on *Helicobacter pylori* eradication in the per-protocol (PP) analysis. Shafaghi et al. had a zero non-eradication cell in the control-arm PP population; Stata’s default 0.5 continuity correction was applied for study-level RR estimation.

Six randomized controlled trials involving 416 participants contributed to the PP analysis. The pooled estimate showed no statistically significant difference in *H. pylori* eradication between adjunctive synbiotic therapy and control treatment (RR 1.06, 95% CI 0.95–1.18; *p* = 0.32). Moderate between-study heterogeneity was observed (*I*^2^ = 48.40%) (Figure 4). In the leave-one-out sensitivity analysis, omission of Shafaghi et al. [26] produced the highest pooled estimate (RR 1.10, 95% CI 0.99–1.23; *p* = 0.086) and reduced heterogeneity from *I*^2^ = 48.40% to 23.75% (Supplementary Figure S2). The lowest pooled estimate was observed after omission of Şirvan et al. (24) (RR 1.02, 95% CI 0.92–1.13). Omission of Tolone et al. [24] yielded a similar non-significant estimate (RR 1.04, 95% CI 0.93–1.18). None of the recalculated estimates were statistically significant, indicating that no individual study materially altered the overall PP findings (Supplementary Figure S3).

### 3.5. Effect of Synbiotic Supplementation on Treatment-Related Adverse Events

**Figure 5:**
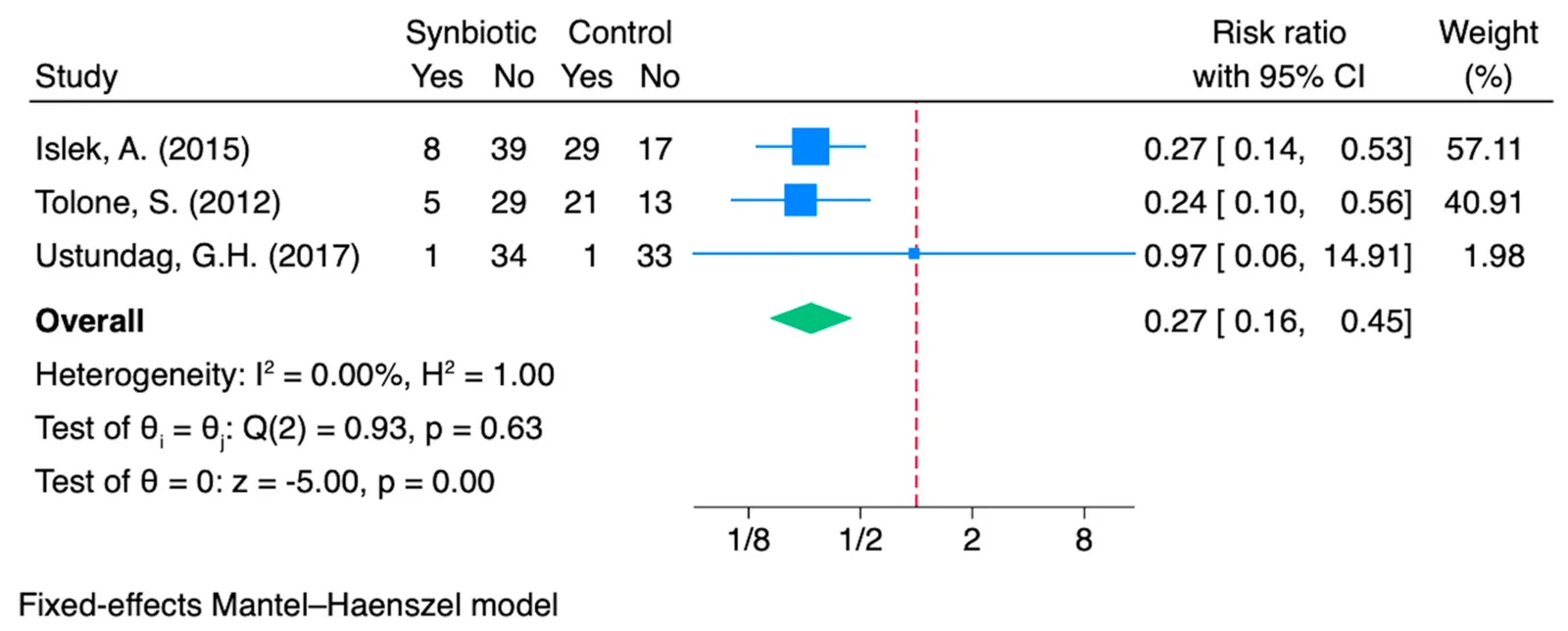
Forest plot of the effect of adjunctive synbiotic supplementation on overall treatment-related adverse events. Risk ratios (RRs) with 95% confidence intervals (CIs) were pooled using a Mantel–Haenszel fixed-effect model. Overall adverse events were defined as participants experiencing at least one treatment-related adverse event during the study period.

Three randomized trials involving 230 participants contributed to the adverse-event analyses. Overall adverse events occurred in 14/116 participants in the synbiotic groups and 51/114 participants in the control groups. The absolute event rates varied across trials. In Islek et al., adverse events occurred in 8/47 participants (17.0%) in the synbiotic group and 29/46 participants (63.0%) in the control group. In Tolone et al., adverse events occurred in 5/34 participants (14.7%) and 21/34 participants (61.8%), respectively. In Ustundag et al., adverse events occurred in 1/35 participants (2.9%) and 1/34 participants (2.9%), respectively. Using a Mantel–Haenszel fixed-effect model, synbiotic supplementation was associated with a lower risk of experiencing at least one adverse event than the control treatment (RR 0.27, 95% CI 0.16–0.45; *p* < 0.001), with no observed heterogeneity (*I*^2^ = 0.00%) (Figure 5). However, the method of ascertainment of adverse events differed between trials, ranging from prospectively completed symptom diaries with prespecified list of symptoms and grading of severity to less intensively described symptom recording (Supplementary Table S3). Thus, the absence of statistical heterogeneity in the relative effects should not be interpreted as indicating similar absolute event frequencies across trials. Therefore, caution should be exercised when interpreting the magnitude of the pooled effect. Symptom-specific adverse events were sparse. Diarrhea occurred in 1 of 116 participants in the synbiotic groups and 13 of 114 participants in the control groups. Nausea and/or vomiting occurred in 3 of 116 and 20 of 114 participants, respectively. These descriptive findings suggest fewer gastrointestinal symptoms with synbiotic supplementation, but the certainty of evidence remains limited because of sparse events and inconsistent adverse-event reporting. These symptom-specific findings were summarized descriptively because of sparse events and inconsistent adverse-event reporting.

### 3.6. Subgroup Analyses

All subgroup analyses were considered exploratory and hypothesis-generating due to the small number of included trials. Several subgroup classifications generated identical or overlapping study partitions and should not be viewed as independent evaluations of effect modification. Specifically, publication period and synbiotic duration generated identical study partitions, while age group, prebiotic composition, and background eradication regimen also generated identical study partitions.

In the ITT analysis, no significant subgroup differences were observed according to publication period, age group, probiotic composition, prebiotic type, treatment duration, or eradication regimen (all *p* values for subgroup difference > 0.05). Higher eradication rates were observed in exploratory subgroups, including pediatric studies, studies using inulin-only formulations, studies using triple therapy (all RR 1.21, 95% CI 1.07–1.36; *I*^2^ = 0.00%), and studies using single-strain probiotics (RR 1.23, 95% CI 1.07–1.42; *I*^2^ = 0%). However, none of the corresponding tests for subgroup differences were statistically significant. Subgroup estimates were also similar between studies published in 2005–2015 and 2016–2024 and between interventions administered for 1 and ≥2 weeks (Table 2 and Supplementary Figure S4).

In the PP analyses, significant subgroup differences were observed according to age group (*p* for interaction = 0.004), probiotic composition (*p* = 0.040), prebiotic composition (*p* = 0.004), and co-intervention regimen (*p* = 0.004). Higher pooled effects were observed in children (RR 1.15, 95% CI 1.03–1.29), single-strain formulations (RR 1.16, 95% CI 1.02–1.31), inulin-only formulations (RR 1.15, 95% CI 1.03–1.29), and studies using triple therapy (RR 1.15, 95% CI 1.03–1.29). No significant subgroup differences were found according to publication period or intervention duration (Table 3 and Supplementary Figure S5). These subgroup findings should be interpreted cautiously because several classifications represented the same underlying study comparisons. The children, inulin-only, and triple therapy subgroups comprised the same four trials, whereas the adult, other-prebiotic, and bismuth-based quadruple-therapy subgroups comprised the same two trials. Moreover, these estimates were strongly influenced by Shafaghi et al. [26], in which differential attrition resulted in a PP control population of 24 participants, all of whom achieved eradication (24/24), compared with 24/38 in the corresponding ITT population.

### 3.7. GRADE Assessment

Using the GRADE approach, the certainty of evidence was rated as low for the ITT eradication outcome, very low for the PP eradication outcome, low for overall adverse events and very low for symptom-specific adverse events. The ITT eradication outcome was downgraded for risk of bias and imprecision. Although the pooled confidence interval excluded the null value, imprecision was considered serious because of the limited sample size and the borderline robustness in the leave-one-out sensitivity analysis. Indirectness was not downgraded because the included populations and eradication regimens were consistent with the prespecified review question, although their applicability to contemporary clinical practice may be limited. The PP outcome was downgraded for risk of bias, inconsistency, and imprecision because of heterogeneity and a confidence interval crossing the null value. Overall adverse events were downgraded for risk of bias because of variability in adverse-event reporting and lack of blinding in some trials, and for indirectness because most safety data were derived from pediatric populations. Diarrhea and nausea and/or vomiting were downgraded for risk of bias, indirectness, and very serious imprecision because events were sparse, some trials reported zero events in one treatment arm, and pooled relative effects were not calculated. Publication bias was not downgraded because the small number of included studies precluded reliable assessment. Detailed GRADE judgments are presented in Table 4.

## 4. Discussion

This updated systematic review and meta-analysis evaluated the effects of synbiotics administered as adjuncts to antibiotic-based *H. pylori* eradication therapy. Across six randomized controlled trials, adjunctive synbiotics were associated with a modest improvement in eradication in the ITT analysis, corresponding to an 18% relative increase compared with eradication therapy alone. However, this benefit was not confirmed in the PP analysis. Synbiotic supplementation was also associated with fewer overall adverse events. For symptom-specific events, diarrhea occurred in 1 of 116 participants receiving synbiotics versus 13 of 114 controls, while nausea and/or vomiting occurred in 3 of 116 versus 20 of 114 participants, respectively. These outcomes were summarized descriptively rather than pooled because event counts were sparse and some trials reported zero events in one treatment arm. The certainty of evidence was low for ITT eradication and overall adverse events, and very low for PP eradication, diarrhea, and nausea and/or vomiting.

These findings partly support those of a previous systematic review specifically examining synbiotics for *H. pylori* eradication, which also reported improved ITT eradication and fewer adverse events, but no significant PP benefit [19]. Similarly, broader meta-analyses of probiotic supplementation have found modest improvements in eradication and treatment tolerability [29,30,31]. However, those reviews pooled heterogeneous probiotic species, doses, treatment durations, antibiotic regimens, and patient populations. Reviews focusing on particular organisms, including *Lactobacillus reuteri* and *Saccharomyces boulardii*, have more consistently demonstrated reductions in gastrointestinal adverse effects than improvements in eradication efficacy [32,33]. Similarly, evidence regarding probiotic supplementation alongside bismuth quadruple therapy suggests a greater benefit for treatment tolerability than for eradication efficacy [34]. Collectively, these findings support the interpretation that the principal clinical benefit of microbial adjuncts may mainly improve treatment completion and tolerability rather than exert a substantial direct effect on *H. pylori* clearance.

The present review provides a methodologically refined update of the previous synbiotic meta-analysis [19]. The search was extended through December 2025, and eligibility was restricted to interventions containing both live microorganisms and prebiotic components in accordance with the current consensus definition of synbiotics [13]. Trials evaluating probiotic-only interventions, prebiotic-only interventions, stand-alone synbiotic treatments, or synbiotics without concomitant antibiotic eradication therapy were excluded. Of the six trials included in the previous review, four met the eligibility criteria of the present review (Shafaghi et al., Şirvan et al., Islek et al., and Ustundag et al.). Gotteland et al. [35] was retrieved and excluded because it did not meet the eligibility criteria, whereas Şahin et al. [23] could not be retrieved. Wang et al. represented the only newly published eligible trial identified after the search period of the previous review, whereas Tolone et al. was additionally identified through citation searching and included in the present review. In addition, ITT and PP outcomes were synthesized separately, the risk of bias was assessed using the RoB 2 tool, and the certainty of evidence was evaluated using the GRADE approach. These methodological refinements reduced the clinical heterogeneity arising from the inclusion of conceptually different interventions and provided a more focused estimate of the effects of adjunctive synbiotic therapy.

The discrepancy between ITT and PP findings is clinically important. ITT analysis estimates the effect of assignment to an intervention while preserving the prognostic balance achieved through randomization and retaining participants who discontinued therapy, were non-adherent, or had missing outcome assessments. By contrast, PP analysis evaluates participants who adequately completed treatment and follow-up and therefore addresses a different clinical question [36]. The significant ITT effect but non-significant PP effect may indicate that synbiotics improve overall treatment effectiveness partly by reducing intolerance and facilitating the completion of antibiotic therapy. Once the analyses were restricted to treatment completers, the additional eradication benefit became smaller and statistically uncertain. Therefore, these results do not establish a strong, direct bactericidal effect of synbiotics. The wide PI crossing the null further indicates that the expected effect may vary across future settings and that the primary ITT findings should be interpreted cautiously given the small number of trials and between-study variability. This interpretation must also consider the risk of bias and clinical context of the contributing trials. In the Shafaghi trial, substantial differential attrition occurred, with considerably more control participants than synbiotic participants excluded from the PP analysis [26]. Although this imbalance may partly reflect poorer treatment tolerance in the control group, differential missing outcome data can exaggerate treatment effects. Conversely, the recent Wang et al. trial found little additional eradication benefit when synbiotics were added to a bismuth quadruple regimen that already achieved a high control-group eradication rate [20]. Tolone et al. had some intervention-reporting ambiguity because the article title referred to lactoferrin and probiotics, whereas the Methods described Probinul probiotic/inulin supplementation [24]. However, exclusion of this trial in sensitivity analysis did not materially alter the overall interpretation of either the ITT or PP findings.

The apparent reduction in the overall adverse events should be interpreted cautiously as ascertainment of adverse events was not standardized across trials. Islek et al. used structured prospective diaries with predefined symptoms and severity grading, whereas Ustundag et al. reported a much lower event frequency despite evaluating a similar pediatric population receiving the same *B. lactis* B94–inulin combination, with less intensively described symptom monitoring. Differences in event solicitation and reporting may have contributed to variation in absolute adverse-event rates across trials despite the absence of statistical heterogeneity in the relative effects. In addition, Islek et al. and Tolone et al. accounted for 98.0% of the pooled weight, indicating that the pooled safety estimate was driven mainly by two trials. The observed reduction in overall adverse events is consistent with previous meta-analyses showing that probiotics may reduce antibiotic-associated gastrointestinal symptoms in adults and children [37,38,39,40]. Therefore, improved tolerability provides a plausible pathway through which synbiotics could improve ITT eradication without producing a clear PP benefit. Nevertheless, the safety evidence was limited. Only three pediatric trials contributed to these analyses, and adverse events were identified using different diaries, questionnaires, or interviews. Some studies reported nausea and vomiting as a combined outcome, whereas others reported these outcomes separately, creating a risk of inconsistent classification or double-counting. Symptom-specific events were sparse, and diarrhea and nausea and/or vomiting were therefore summarized descriptively rather than pooled. Therefore, the safety results should not be interpreted as establishing a consistent protective effect across all formulations, age groups, or eradication regimens.

Several biological mechanisms could contribute to the improved tolerability or adjunctive antimicrobial activity observed with synbiotic supplementation. Selected probiotic organisms can produce lactic acid, short-chain fatty acids, bacteriocins, and other metabolites capable of inhibiting *H. pylori* growth [41,42,43,44,45]. Other strains may interfere with bacterial adhesion to gastric epithelial cells, compete for nutrients or binding sites, strengthen epithelial-barrier function, and attenuate *H. pylori*-induced inflammatory signaling [46,47]. Prebiotic substrates, such as inulin, fructooligosaccharides, and resistant dextrin, may promote the activity of beneficial resident microorganisms or support the activity of the administered probiotic component [13]. However, these effects are strain- and substrate-specific. Because the included trials used different single- and multi-strain products and did not directly compare probiotic-only, prebiotic-only, and synbiotic interventions, the present analysis cannot determine whether the observed benefits resulted from the probiotic strains, the prebiotic substrates, their interactions, or improved adherence. Thus, the available evidence does not establish the superiority of any specific probiotic strain or prebiotic component. Although the PP subgroup analysis suggested greater effects with single-strain and inulin-only formulations, these findings should be interpreted cautiously because of the small number of trials and substantial overlap among subgroup classifications. Preservation of intestinal microbiota may represent an additional benefit. *H. pylori* eradication therapy can substantially disrupt intestinal microbial diversity and composition, with some alterations persisting after treatment. Randomized studies have suggested that selected probiotic interventions may attenuate antibiotic-associated microbiome disruption or accelerate microbial recovery [48,49,50]. In the Wang trial, synbiotic supplementation helped maintain aspects of microbial diversity during bismuth quadruple therapy, despite providing no apparent eradication advantage [20]. This supports a potential microbiome-protective effect, although its durability and clinical relevance remain uncertain.

Differences in participant age, geographical setting, synbiotic composition, treatment duration, antibiotic regimen, baseline eradication success, and local antimicrobial resistance likely contributed to the heterogeneity. No significant subgroup differences were detected in the ITT analysis. In the PP analysis, significant subgroup differences were observed according to age group, probiotic composition, prebiotic composition, and background eradication regimen. These results should be considered exploratory because several subgroup categories contained identical or substantially overlapping sets of studies. Specifically, the pediatric, inulin-only, and triple therapy subgroups comprised the same four trials, whereas the adult, other-prebiotic, and bismuth quadruple therapy subgroups comprised the same two trials. Credible subgroup effects generally require prespecification, formal interaction testing, biological plausibility, consistency, and an adequate number of studies and participants [51,52]. Therefore, the current evidence does not demonstrate that age group, single-strain products, inulin-containing formulations, or triple therapy independently modified the effect of adjunctive synbiotics.

Generalizability is limited by changes in the treatment context. Four of the six trials enrolled children, and most pediatric studies used clarithromycin-containing triple therapy without reporting antimicrobial susceptibility. Global analyses have documented substantial geographical variation and increasing resistance to clarithromycin and metronidazole, including among pediatric populations [53,54,55]. Clarithromycin resistance now exceeds the thresholds at which empirical clarithromycin-based triple therapy is discouraged in many settings [56]. Therefore, current adult and pediatric guidelines favor optimized bismuth quadruple therapy or susceptibility-guided therapy, when appropriate [57,58]. This changing treatment context may partly explain why the updated pooled ITT effect was smaller than that reported in the previous review. In particular, the Wang trial may represent a ceiling effect, in which an adjunctive intervention has limited opportunity to improve eradication when added to an already highly effective bismuth quadruple regimen. Therefore, synbiotic supplementation should not be used to compensate for an eradication regimen that is unlikely to be effective because of local antimicrobial resistance.

Several limitations should be acknowledged. First, only six relatively small randomized controlled trials were included, limiting the statistical power and precision of the pooled estimates. Second, the synbiotic formulations, doses, treatment durations, participant populations, and antibiotic regimens differed across studies. Third, one trial was judged to be at high overall risk of bias, and the remaining five raised some concerns; none was rated as having a low overall risk of bias. Fourth, safety outcomes were reported by few studies, and symptom-specific adverse events were not reported in a sufficiently comparable manner to permit pooling. The limitations of the review process should also be considered. Publication bias could not be formally assessed because fewer than 10 studies were available for each meta-analysis. In addition, subgroup analyses were based on a small number of aggregate study-level comparisons and were vulnerable to ecological confounding. These limitations should be distinguished from those of the primary studies themselves, in accordance with PRISMA guidance [21].

## 5. Conclusions

Adjunctive synbiotic therapy may modestly improve *H. pylori* eradication in ITT analyses and may reduce overall treatment-related adverse events. However, evidence for individual gastrointestinal adverse events remains limited because symptom-specific events were sparse and were not quantitatively pooled. These findings suggest that synbiotics may primarily improve treatment tolerability and completion rather than exert a substantial direct eradication effect. Therefore, synbiotics should be considered only as adjuncts and not as substitutes for guideline-recommended antibiotic therapy. Given the low-to-very-low certainty of evidence across the assessed outcomes, no specific probiotic strain, prebiotic component, dose, duration, or eradication regimen can currently be recommended. Further adequately powered, prospectively registered trials using clearly characterized synbiotic formulations, rigorous randomization methods, standardized adverse-event reporting, objective adherence assessment, and longer follow-up are needed to determine their effects on eradication, recurrence, microbiome recovery, and antimicrobial resistance.

## Figures and Tables

**Table 1:** Characteristics of included studies.

First Author	Year	Country	Sample Size (*n*)	Mean Age (Years)	Sex (M:F)	Baseline *H. pylori* Diagnostic Method	Post-Treatment Eradication Assessment	Study Duration	Probiotic Strains	Prebiotic Component	Duration of Synbiotic Use	Eradication Regimen Co-Intervention
Islek, A. [25]	2015	Turkey	93	11.95 ± 2.69	38:55	RUT, Histology, Stool antigen	Stool antigen test (6–8 weeks after completion of lansoprazole treatment)	7–9 weeks	*B. lactis* B94	Inulin	7 days	Triple therapy
Shafaghi, A. [26]	2016	Iran	76	43.50 ± 13.31	35:41	Histology, RUT	^14C-UBT (8 weeks after completion of treatment)	10 weeks	*L. casei*, *L. rhamnosus*, *S. thermophilus*, *B. breve*, *L. acidophilus*, *B. longum*, *L. bulgaricus*	FOS	14 days	Bismuth quadruple therapy
Şirvan, B.N. [27]	2017	Turkey	100	12.05 ± 3.45	45:55	Histology	Stool antigen test (1 month after completion of eradication treatment)	6 weeks	*B. lactis*	Inulin	14 days	Triple therapy
Tolone, S. [24] †	2012	Italy	68	8.30 ± 3.40	32:36	Histology, ^13C-UBT	^13C-UBT (4 weeks after discontinuation of therapy)	5 weeks	*L. plantarum*, *L. reuteri*, *L. casei subsp. rhamnosus*, *B. infantis*, *B. longum*, *L. salivarius*, *L. acidophilus*, *S. thermophilus*, *L. sporogenes*	Inulin	7 days	Triple therapy
Ustundag, G.H. [28]	2017	Turkey	69	11.20 ± 3.00	33:36	Histology	^14C-UBT (4–6 weeks after cessation of omeprazole treatment)	6–8 weeks	*B. lactis* B94	Inulin	14 days	Triple therapy
Wang, H. [20]	2024	China	80	39.30 ± 12.38	34:46	^13C-UBT, Gastric biopsy	^13C-UBT (week 6 after initiation of intervention; approximately 4 weeks after completion of quadruple therapy)	6 weeks	*B. animalis BB12*, *B. lactis Bi07*, *B. bifidum Bb06*, *L. helveticus R52*, *L. rhamnosus R11*, *L. acidophilus NCFM*	Dextrin, FOS, Inulin	6 weeks	Bismuth quadruple therapy

Footnote: M:F, male-to-female ratio; RUT, rapid urease test; ^13C-UBT, ^13C-urea breath test; ^14C-UBT, ^14C-urea breath test; FOS, fructooligosaccharides. Wang et al. randomized 80 participants across four arms; only the QT (*n* = 20) and QS (*n* = 20) arms were included in the present meta-analysis. † Probinul was described in the Methods as containing inulin; therefore, the prebiotic component was coded as inulin.

**Table 2:** Subgroup analyses of the intention-to-treat (ITT) *Helicobacter pylori* eradication outcome.

Category	Subgroup	Studies (*n*)	Participants (*n*)	RR	95% CI	*I*^2^ (%)	*p*
Publication period **^†^**	2005–2015	2	161	1.17	1.00–1.38	0.00	0.893
	2016–2024	4	285	1.20	0.98–1.45	64.26	
Age group **^‡^**	Adult	2	116	1.16	0.76–1.77	86.14	0.865
	Children	4	330	1.21	1.07–1.36	0.00	
Probiotic composition	Mixed	3	184	1.15	0.90–1.47	73.09	0.644
	Single	3	262	1.23	1.07–1.42	0.00	
Prebiotic composition **^‡^**	Inulin-only	4	330	1.21	1.07–1.36	0.00	0.865
	Other	2	116	1.16	0.76–1.77	86.14	
Synbiotic duration **^†^**	1 wk	2	161	1.17	1.00–1.38	0.00	0.893
	≥2 wks	4	285	1.20	0.98–1.45	64.26	
Eradication regimen **^‡^**	Quadruple therapy	2	116	1.16	0.76–1.77	86.14	0.865
	Triple therapy	4	330	1.21	1.07–1.36	0.00	

Footnote: CI, confidence interval; RR, risk ratio. The *p* value for subgroup differences represents the test for interaction between subgroups. All subgroup analyses were exploratory and hypothesis-generating. The 2005–2015 category represents the prespecified early-publication stratum; the earliest included trial in this stratum was published in 2012. Several subgroup variables generated identical or overlapping study partitions. ^†^ Publication period and synbiotic duration generated identical study partitions. ^‡^ Age group, prebiotic composition, and eradication regimen generated identical study partitions. Therefore, these findings should not be interpreted as independent evidence of effect modification.

**Table 3:** Subgroup analyses of the per-protocol (PP) *Helicobacter pylori* eradication outcome.

Category	Subgroup	Studies (*n*)	Participants (*n*)	RR	95% CI	*I*^2^ (%)	*p*
Publication period **^†^**	2005–2015	2	150	1.10	0.95–1.27	0.00	0.670
	2016–2024	4	266	1.05	0.90–1.23	64.58	
Age group **^‡^**	Adult	2	102	0.93	0.85–1.03	0.00	0.004
	Children	4	314	1.15	1.03–1.29	0.00	
Probiotic composition	Mixed	3	170	0.97	0.88–1.08	21.67	0.040
	Single	3	246	1.16	1.02–1.31	0.00	
Prebiotic composition **^‡^**	Inulin-only	4	314	1.15	1.03–1.29	0.00	0.004
	Other	2	102	0.93	0.85–1.03	0.00	
Synbiotic duration **^†^**	1 wk	2	150	1.10	0.95–1.27	0.00	0.67
	≥2 wks	4	266	1.05	0.90–1.23	64.58	
Eradication regimen **^‡^**	Quadruple therapy	2	102	0.93	0.85–1.03	0.00	0.004
	Triple therapy	4	314	1.15	1.03–1.29	0.00	

Footnote: CI, confidence interval; RR, risk ratio. The *p* value for subgroup differences represents the statistical test for the interaction between categories within each category. All subgroup analyses were exploratory and hypothesis-generating. The 2005–2015 category represents the prespecified early-publication stratum; the earliest included trial in this stratum was published in 2012. Several subgroup variables generated identical or overlapping study partitions. ^†^ Publication period and synbiotic duration generated identical study partitions. ^‡^ Age group, prebiotic composition, and eradication regimen generated identical study partitions. Therefore, these findings should not be interpreted as independent evidence of effect modification.

**Table 4:** Summary of findings and GRADE assessments for adjunctive synbiotic therapy during *Helicobacter pylori* eradication therapy.

Outcome	Studies (Participants)	Events/ Participants, Synbiotic vs. Control	Relative Effect (95% CI)	Anticipated Absolute Effect Per 1000 Participants	Risk of Bias	Inconsistency	Indirectness	Imprecision	Publication Bias	Certainty of Evidence
*H. pylori* eradication (ITT)	6 (446)	192/224 vs. 156/222	RR 1.18 (1.04–1.34)	126 more eradications (27 to 239 more)	Serious	Not serious	Not serious	Serious	Not downgraded	⨁⨁◯◯ Low
*H. pylori* eradication (PP)	6 (416)	192/219 vs. 156/197	RR 1.06 (0.95–1.18)	48 more eradications (40 fewer to 143 more)	Serious	Serious	Not serious	Serious	Not downgraded	⨁◯◯◯ Very low
Overall adverse event	3 (230)	14/116 vs. 51/114	RR 0.27 (0.16–0.45)	327 fewer adverse events (376 to 246 fewer)	Serious	Not serious	Serious	Not serious	Not downgraded	⨁⨁◯◯ Low
Diarrhea	3 (230)	1/116 vs. 13/114	Not pooled	Not estimated	Serious	Not Serious	Serious	Very serious	Not downgraded	⨁◯◯◯ Very low
Nausea and/or vomiting	3 (230)	3/116 vs. 20/114	Not pooled	Not estimated	Serious	Not serious	Serious	Very serious	Not downgraded	⨁◯◯◯ Very Low

Footnote: CI, confidence interval; GRADE, Grading of Recommendations Assessment, Development and Evaluation; ITT, intention-to-treat; PP, per-protocol; RR, risk ratio. Symptom-specific adverse events were not pooled because event counts were sparse and some trials reported zero events in one treatment arm. Publication bias was not downgraded because the small number of included studies precluded reliable assessment; therefore, this judgment should not be interpreted as evidence of absence of publication bias. GRADE certainty definitions: High certainty (⨁⨁⨁⨁), the true effect is likely to be close to the estimated effect; moderate certainty (⨁⨁⨁◯), the true effect is probably close to the estimated effect but may differ substantially; low certainty (⨁⨁◯◯), the true effect may differ substantially from the estimated effect; and very low certainty (⨁◯◯◯), the true effect is likely to differ substantially from the estimated effect.

## Data Availability

All data analyzed during this study are included in this published article and its Supplementary Materials. Further clarifications may be obtained from the corresponding author upon reasonable request.

## Supplementary Materials

The following supporting information can be downloaded at: https://www.mdpi.com/article/10.3390/life16091409/s1, Figure S1: Leave-one-out sensitivity analysis for the intention-to-treat eradication outcome; Figure S2: Forest plot of the per-protocol eradication analysis after excluding Shafaghi et al.; Figure S3: Leave-one-out sensitivity analysis for the per-protocol eradication outcome; Figure S4: Subgroup analyses of the effect of adjunctive synbiotic therapy on *H. pylori* eradication in the intention-to-treat population; Figure S5: Subgroup analyses of the effect of adjunctive synbiotic therapy on *H. pylori* eradication in the per-protocol population; Table S1: Database-specific search strategies; Table S2: RoB 2 signalling questions and domain-level risk-of-bias judgments for the included randomized controlled trials; Table S3: Adverse-event ascertainment and reporting methods across the included trials; Dataset S1: Extracted study-level dataset used for the intention-to-treat, per-protocol, and adverse-event analyses.

## Abbreviations

The following abbreviations are used in this manuscript:

CI	Confidence interval
FOS	Fructo-oligosaccharide
GRADE	Grading of Recommendations Assessment, Development and Evaluation
*H. pylori*	*Helicobacter pylori*
ITT	Intention-to-treat
ISAPP	International Scientific Association for Probiotics and Prebiotics
*I*^2^	Inconsistency statistic
PI	Prediction interval
PRISMA	Preferred Reporting Items for Systematic Reviews and Meta-Analyses
PROSPERO	International Prospective Register of Systematic Reviews
PP	Per-protocol
PPI	Proton pump inhibitor
QT	Quadruple therapy
QS	Quadruple therapy plus synbiotic
RCT	Randomized controlled trial
REML	Restricted maximum likelihood
RoB 2	Revised Cochrane risk-of-bias tool for randomized trials
RR	Risk ratio
RUT	Rapid urease test
UBT	Urea breath test
^13C-UBT	Carbon-13 urea breath test
^14C-UBT	Carbon-14 urea breath test
