# Supplementary material for: Adjunctive Synbiotic Therapy for Helicobacter pylori Eradication: An Updated Systematic Review and Meta-Analysis of Randomized Controlled Trials
Source: Life (Basel). 2026 Aug 25;16(9):1409. doi: 10.3390/life16091409 (PMC13608589; doi:10.3390/life16091409)
Supplement: Supplementary file 1 [file life-16-01409-s001.zip › 20260818 Supplementary figure.pdf]

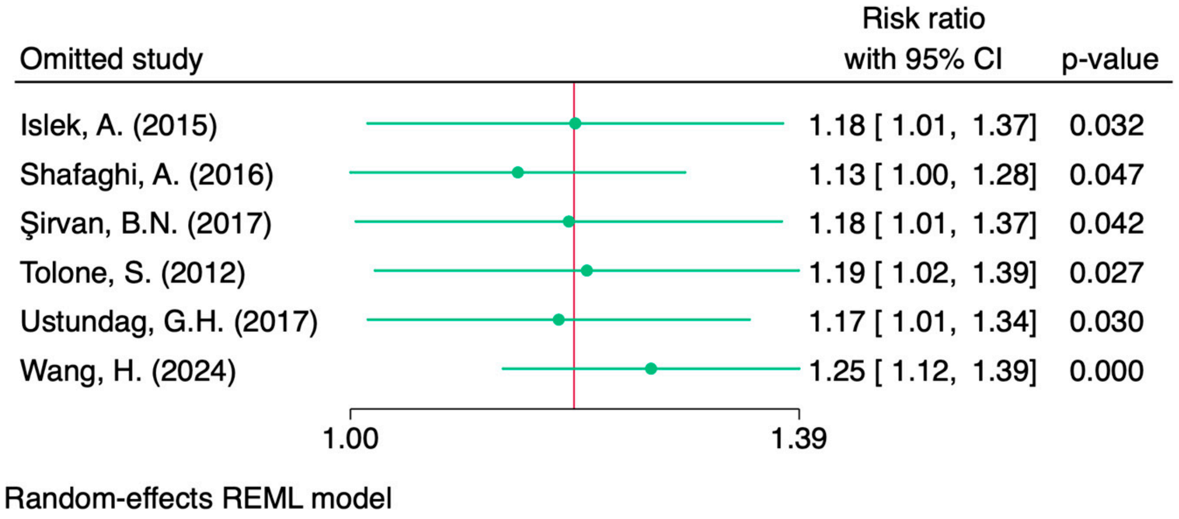

Figure S1. Leave-one-out sensitivity analysis of the intention-to-treat eradication outcome.

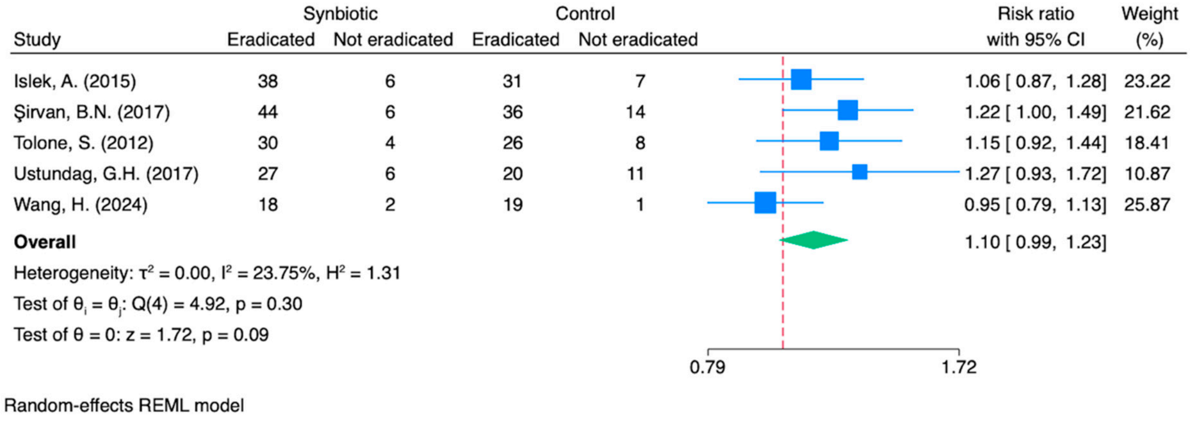

Figure S2. Forest plot of the per-protocol eradication analysis after excluding Shafaghi et al.

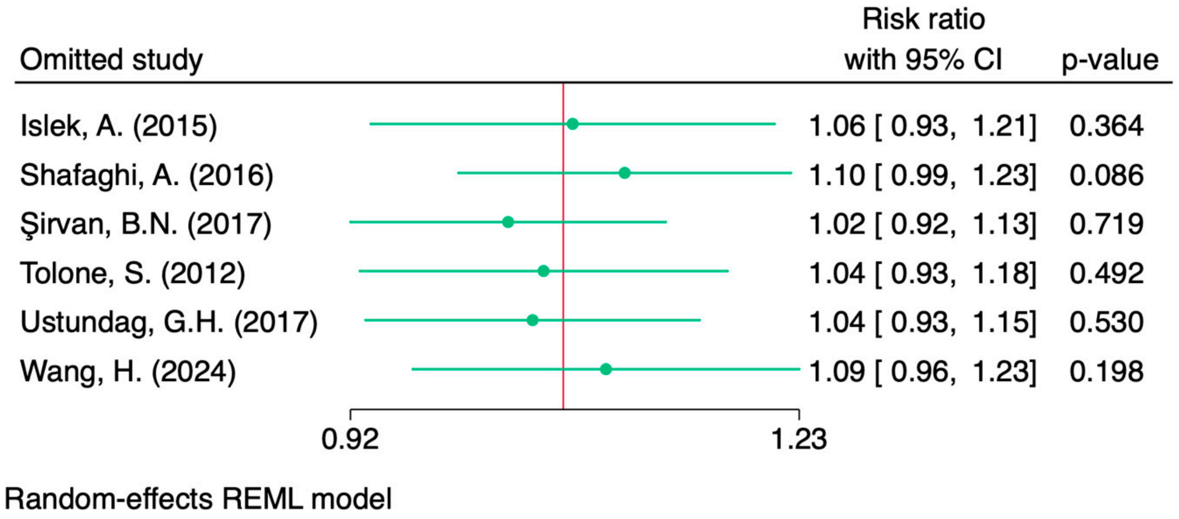

Figure S3. Leave-one-out sensitivity analysis of the per-protocol eradication outcome.

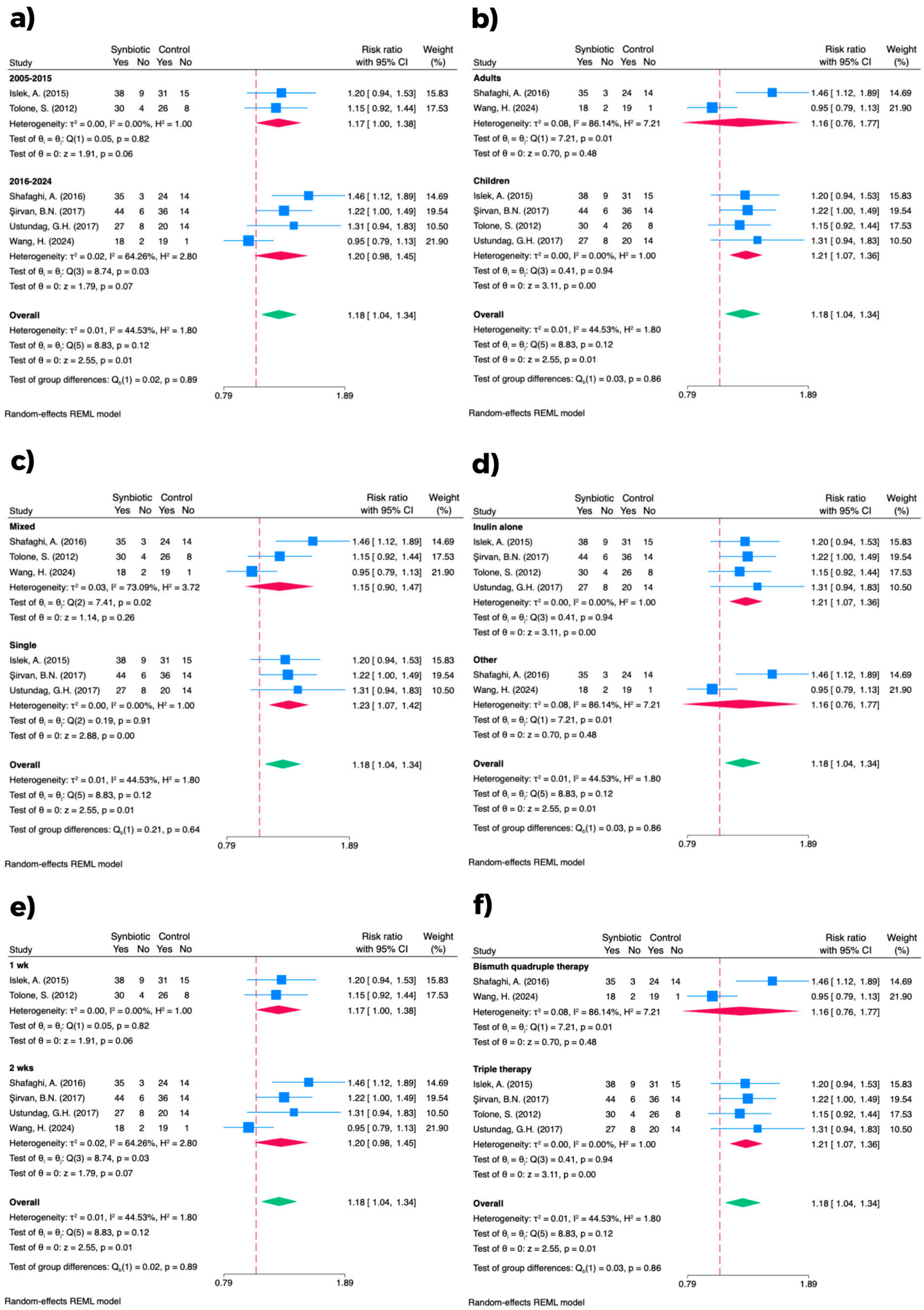

Figure S4. Subgroup analyses of the effect of adjunctive synbiotic therapy on *H. pylori* eradication in the intention-to-treat population. Subgroup analyses were conducted according to **a)** publication period, **b)** age group, **c)** probiotic composition, **d)** prebiotic composition, **e)** duration of synbiotic administration, and **f)** background eradication regimen.

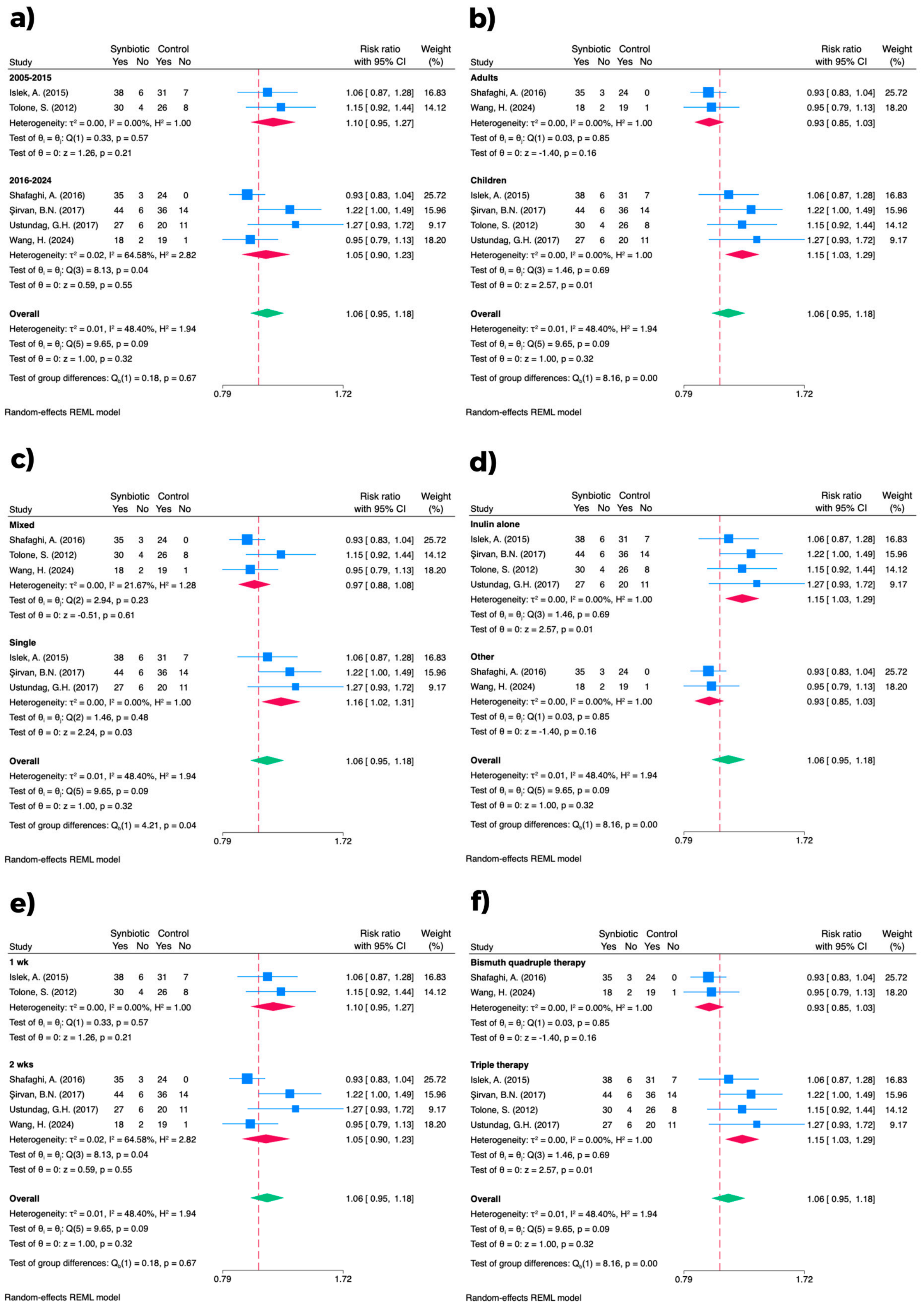

Figure S5. Subgroup analyses of the effect of adjunctive synbiotic therapy on *H. pylori* eradication in the per-protocol population. Subgroup analyses were conducted according to **a)** publication period, **b)** age group, **c)** probiotic composition, **d)** prebiotic composition, **e)** duration of synbiotic administration, and **f)** background eradication regimen.
