# Supplementary material for: Adjunctive Synbiotic Therapy for Helicobacter pylori Eradication: An Updated Systematic Review and Meta-Analysis of Randomized Controlled Trials
Source: Life (Basel). 2026 Aug 25;16(9):1409. doi: 10.3390/life16091409 (PMC13608589; doi:10.3390/life16091409)
Supplement: Supplementary file 1 [file life-16-01409-s001.zip › 20260818 Supplementary Table .pdf]

Supplementary Table S1. Database-specific search strategies

Database	Search date	Final search strategy	Records retrieved
PubMed	14 November 2025	("Helicobacter pylori"[MeSH Terms] OR "Helicobacter pylori"[Title/Abstract] OR "H. pylori"[Title/Abstract] OR "Helicobacter"[MeSH Terms] OR Helicobacter[Title/Abstract]) AND ("Synbiotics"[MeSH Terms] OR synbiotic* [Title/Abstract] OR "syn-biotic"[Title/Abstract] OR "probiotic-prebiotic"[Title/Abstract] OR "combined probiotic"[Title/Abstract] OR (probiotic* [Title/Abstract] AND prebiotic*[Title/Abstract]))	78
Scopus	14 November 2025	(TITLE-ABS-KEY("Helicobacter pylori") OR TITLE-ABS-KEY("H. pylori") OR TITLE-ABS-KEY(Helicobacter)) AND (TITLE-ABS-KEY(synbiotic*) OR TITLE-ABS-KEY("syn-biotic") OR TITLE-ABS-KEY("probiotic prebiotic") OR TITLE-ABS-KEY("combined probiotic") OR (TITLE-ABS-KEY(probiotic*) AND TITLE-ABS-KEY(prebiotic*)))	417
Embase	14 November 2025	(exp Helicobacter pylori/ OR "helicobacter pylori".mp. OR "h. pylori".mp.) AND (exp symbiotic agent/ OR synbiotic*.mp. OR "probiotic prebiotic combination".mp. OR ((exp probiotic agent/ OR probiotic*.mp.) AND (exp prebiotic agent/ OR prebiotic*.mp.)))	285
MEDLINE	14 November 2025	(exp Helicobacter pylori/ OR "helicobacter pylori".mp. OR exp Helicobacter Infections/ OR "h pylori".mp.) AND (exp Synbiotics/ OR synbiotic.mp. OR "probiotic prebiotic".mp. OR ((exp Probiotics/ OR probiotic.mp.) AND (exp Prebiotics/ OR prebiotic.mp.)))	33
Web of Science	19 December 2025	TS=("Helicobacter pylori" OR "H.pylori" OR Helicobacter) AND TS=(synbiotic* OR "syn-biotic*" OR "probiotic-prebiotic" OR "combined probiotic*" OR (probiotic* AND prebiotic*))	173
Google Scholar	19 December 2025	Synbiotic and Helicobacter pylori eradication	First 210 records
Total			1,196

Abbreviations: MeSH, Medical Subject Headings; mp, multipurpose field; TITLE-ABS-KEY, title, abstract, and keywords; TS, topic search.

Supplementary Table S2. RoB 2 signalling questions and domain-level risk-of-bias judgments for the included randomized controlled trials

RoB 2 signalling question	Islek et al.	Shafaghi et al.	Şirvan et al.	Tolone et al.	Ustundag et al.	Wang et al.
**D1. Bias arising from the randomization process**						
1.1 Was the allocation sequence random?	PY	PY	PY	PY	PY	Y
1.2 Was the allocation sequence concealed until participants were enrolled and assigned?	PY	NI	NI	NI	NI	NI
1.3 Did baseline differences between intervention groups suggest a problem with randomization?	N	N	N	N	N	N
**Domain 1 judgment**	**Low**	**Some concerns**	**Some concerns**	**Some concerns**	**Some concerns**	**Some concerns**
**D2. Bias due to deviations from intended interventions**						
2.1 Were participants aware of their assigned intervention?	N	N	Y/PY	Y/PY	NI	Y
2.2 Were carers/people delivering the intervention aware?	N	N	Y/PY	Y/PY	NI	Y
2.3 Were there deviations from intended intervention because of the trial context?	NA	NA	N	N	N	N
2.4 Were such deviations likely to have affected the outcome?	NA	NA	NA	NA	NA	NA
2.5 Were such deviations balanced between groups?	NA	NA	NA	NA	NA	NA
2.6 Was an appropriate analysis used to estimate the effect of assignment to intervention?	Y	Y	Y	Y	Y	Y
2.7 Could failure to analyse participants in their randomized groups substantially affect the result?	NA	NA	NA	NA	NA	NA
**Domain 2 judgment**	**Low**	**Low**	**Low**	**Low**	**Low**	**Low**
**D3. Bias due to missing outcome data**						
3.1 Were outcome data available for all, or nearly all, randomized participants?	PN	N	Y	Y	PY	Y
3.2 Is there evidence that the result was not biased by missing outcome data?	PY	N/PN	NA	NA	PY	NA
3.3 Could missingness depend on the true value of the outcome?	PY	Y	NA	NA	PY	NA

RoB 2 signalling question	Islek et al.	Shafaghi et al.	Şirvan et al.	Tolone et al.	Ustundag et al.	Wang et al.
3.4 Is it likely that missingness depended on **the true value of the outcome?**	PN	Y/PY	NA	NA	PN	NA
**Domain 3 judgment**	**Some concerns**	**High**	**Low**	**Low**	**Some concerns**	**Low**
**D4. Bias in measurement of the outcome**						
4.1 Was the method of measuring the outcome inappropriate?	N	N	N	N	N	N
4.2 Could measurement or ascertainment have differed between intervention groups?	N	N	N	N	N	N
4.3 Were outcome assessors aware of the intervention received?	NI	NI	NI	NI	NI	Y/NI
4.4 Could assessment of the outcome have been influenced by knowledge of intervention received?	N	N	N	N	N	N
4.5 Is it likely that assessment was influenced by knowledge of intervention received?	NA	N	N	N	N	N
**Domain 4 judgment**	**Low**	**Low**	**Low**	**Low**	**Low**	**Low**
**D5. Bias in selection of the reported result**						
5.1 Were the data analysed according to a prespecified analysis plan finalized before unblinded outcome data were available?	NI	NI	NI	NI	NI	NI
5.2 Is the numerical result likely to have been selected from multiple eligible outcome measurements?	PN	PN	PN	PN	PN	PN
5.3 Is the numerical result likely to have been selected from multiple eligible analyses?	PN	PN	PN	PN	PN	PN
**Domain 5 judgment**	**Some concerns**	**Some concerns**	**Some concerns**	**Some concerns**	**Some concerns**	**Some concerns**
**Overall risk of bias**	**Some concerns**	**High**	**Some concerns**	**Some concerns**	**Some concerns**	**Some concerns**

Footnote: Y, yes; PY, probably yes; PN, probably no; N, no; NI, no information; NA, not applicable. RoB 2 assessments targeted the effect of assignment to intervention for the intention-to-treat *H. pylori* eradication outcome.

Supplementary Table S3. Adverse-event ascertainment and reporting methods across the included trials

Study	Adverse-event ascertainment method	Timing/follow-up	Adverse events assessed/reported	Comments on ascertainment
Islek et al.	Prospective patient/parent diary with prespecified symptoms and severity grading	During treatment	Eructation, nausea/vomiting, abdominal/epigastric pain, taste disturbance, diarrhea	Structured active symptom solicitation
Shafaghi et al.	Study questionnaires assessing frequency and severity of treatment-related adverse effects	At baseline and during treatment	Flatulence, taste abnormality, epigastric pain, nausea/vomiting, constipation, diarrhea, anorexia	Structured questionnaire-based assessment
Şirvan et al.	Patient/parent diary recording compliance, symptoms, and side effects	During treatment with telephone and clinical follow-up	Epigastric pain, nausea/vomiting, metallic taste, diarrhea	Prospective diary with active follow-up
Tolone et al.	Parent diary with predefined list of potential adverse effects	During treatment	Epigastric pain, nausea, vomiting, diarrhea, constipation	Structured prospective symptom solicitation
Ustundag et al.	Patient/parent symptom and adverse-effect recording form	During treatment	Nausea, vomiting, diarrhea, abdominal/epigastric pain	Ascertainment procedures described less extensively than in Islek et al.
Wang et al.	New-onset symptoms recorded during study	During 6-week study period	New-onset symptoms	Limited detail on structured symptom solicitation
